# Achieving Robust Single-Photon Blockade with a Single Nanotip

**DOI:** 10.1021/acs.nanolett.4c05433

**Published:** 2025-03-04

**Authors:** Jian Tang, Yunlan Zuo, Xun-Wei Xu, Ran Huang, Adam Miranowicz, Franco Nori, Hui Jing

**Affiliations:** † Key Laboratory of Low-Dimensional Quantum Structures and Quantum Control of Ministry of Education, Department of Physics and Synergetic Innovation Center for Quantum Effects and Applications, Hunan Normal University, Changsha 410081, China; ‡ School of Physics and Chemistry, Hunan First Normal University, Changsha 410205, China; ¶ Quantum Information Physics Theory Research Team, Quantum Computing Center, RIKEN, Wako-shi, Saitama 351-0198, Japan; § Institute of Spintronics and Quantum Information, Faculty of Physics and Astronomy, Adam Mickiewicz University, 61-614 Poznań, Poland; ∥ Physics Department, The University of Michigan, Ann Arbor, Michigan 48109-1040, United States; ⊥ Institute for Quantum Science and Technology, College of Science, National University of Defense Technology, Changsha 410073, P.R.China

**Keywords:** backscattering loss, photon blockade, nanotip, quantum correlation, single photon, optical nonlinearity

## Abstract

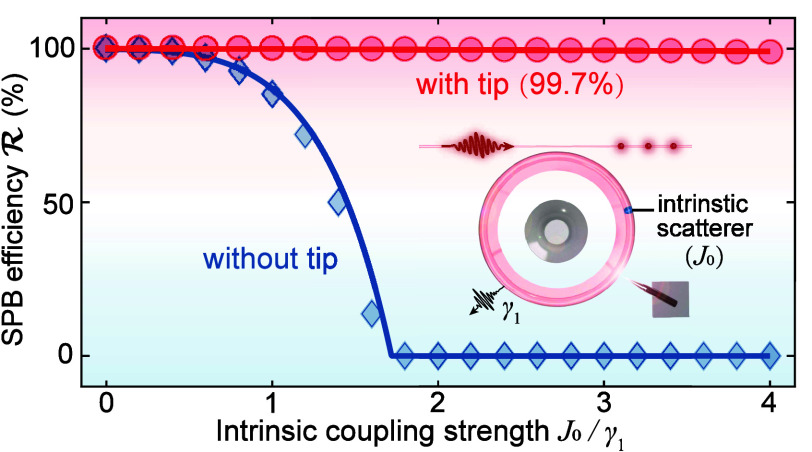

Backscattering losses (BSL), arising from intrinsic imperfections or unavoidable external perturbations in optical resonators, can severely impact photonic devices. In single-photon systems, robust quantum correlations against BSL remain largely unexplored despite their significance for various applications. Here, we demonstrate that single-photon blockade (SPB), a purely quantum effect, can be preserved against BSL by introducing a nanotip near a Kerr nonlinear resonator with intrinsic defects. Without the tip, BSL disrupts SPB, but tuning the tip’s position restores robustness even under strong BSL. Notably, quantum correlations emerge while the classical mean photon number remains suppressed due to the interplay between resonator nonlinearity and tip-induced optical coupling. Our findings highlight nanoscale engineering as a powerful tool to protect and harness fragile quantum correlations, paving the way for robust single-photon sources and backscattering-immune quantum devices.

Single-photon quantum optics is pivotal for advancing quantum information technologies, enabling applications such as quantum communications,^1−3^ quantum computing,^4−7^ and quantum optical metrology.^8^ In this context, single-photon blockade (SPB),^9−12^ indicating blockade of the subsequent photons by absorbing the first one, goes beyond classical optics and laser physics into a purely quantum regime. Due to its crucial role in generating nonclassical correlations and constructing single-photon devices, SPB has been demonstrated experimentally in various systems ranging from micro- or nanoscale cavities with atoms,^13−15^ quantum dots,^16−20^ or superconducting qubits,^21−25^ to cavity-free atoms^26,27^ or Bose-Hubbard chains.^28^ In addition, multiphoton blockade^29−31^ has recently been observed, opening the way to create few-photon devices for quantum networks.

Optical whispering-gallery-mode (WGM) microresonators are excellent platforms for achieving SPB due to their ability to confine light in a circular path within a microscale volume, leading to strongly enhanced light-matter interactions. These microresonators are not only significant for fundamental studies in nonlinear optics^32,33^ but also play a crucial role in nano-optics applications, particularly ultrasensitive nanoscale sensing.^34−38^ However, In a real WGM cavity, imperfections—like intrinsic material defects, density variations, or surface roughness—can cause backscattered light in the counter-propagating direction, leading to an extra optical loss and mode coupling. Such backscattering has been used to realize counter-propagating solitons,^39^ chiral lasing,^40^ absorption^41^ and topological,^42^ as well as slow light and its localization.^43^ However, backscattering loss limits the application performance in classical and quantum devices, such as instability problems in frequency combs,^44,45^ backscattering-induced noise, and lock-in effect in optical gyroscopes,^46−48^ as well as decrease of secure key rates in quantum key distribution.^49,50^

To overcome these challenges, backscattering loss suppression was experimentally studied by introducing reflectors or scatterers, ranging from macroscale mirrors^51,52^ to Mie^53^ and Rayleigh^54^ scatterers. Also, Brillouin scattering,^55,56^ active feedback control,^57^ self-injection technique,^58^ and synthetic gauge fields^59^ were used to suppress backscattering. These remarkable achievements provide powerful tools to optimize optical devices^56^ and explore nonreciprocal optics^59^ or non-Hermitian physics.^60^ Yet previous efforts have been devoted to propagation against backscattering loss of *many photons or classical light*,^51−57,59−62^ it is essential to study robust nonclassical single-photon effects in spite of intrinsic defects, which are expected to play a key role in realistic single-photon devices and quantum technologies.

In this letter, we study the realization of *robust quantum correlation of single photons* against backscattering loss via the SPB effect in nonlinear WGM cavities by introducing a nanotip. Although, backscattering loss can lead to the breakdown of SPB in conventional WGM cavities, we find robust SPB can be revived with an efficiency of up to 99.7% by precisely tuning the position of the nanotip, and it is robust with different backscattering strengths. Different from the behavior of classical mean photon number, SPB can emerge when the mean photon number is still suppressed, since our findings do not merely rely on the nanotip-induced destructive interference,^51−54^ but on the *interplay* of the resonator nonlinearity and the nanotip-induced optical coupling. Instead of analyzing light amplitudes,^51−57,59,60^ we focus on quantum correlations and the transitions between quantum states, which hold the potential for implementations in quantum information technologies. Our findings drive the field of backscattering suppression into the quantum regime, hence making it possible to realize a variety of quantum backscattering-immune effects, such as multiphoton blockade against backscattering loss^29−31^ or one-way single-photon transmission,^63,64^ for potential applications of nanoscale engineering in robust quantum devices and the protection of fragile quantum resources.

We consider an optical Kerr resonator with an additional nanotip [Figure 1(a)]. For an ideal cavity driven from the left-hand side, only the clockwise (CW) mode is dominant. In a real cavity, intrinsic defects cause backscattering in the counterclockwise (CCW) direction, which can be approximated as an effective single scatterer,^54,65^ leading to the coupling between the CW and CCW modes (with strength *J*_0_).^66−68^ We note that the intrinsic backscattering strength is proportional to *J*_0_.^69−71^ Introducing a nanotip also leads to coupling between the two modes, which can in general be nonsymmetric leading to the studies of non-Hermitian physics with exceptional points.^53,66,72−74^ However, such couplings are typically assumed to be equal (i.e., without exceptional points).^41,75,76^ Thus, the total optical coupling can be written as (ℏ = 1):
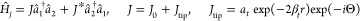
1Here, *â*_1_ (*â*_2_) is the annihilation operator for the CW (CCW) mode, *J*_tip_ is the tip-induced coupling strength with amplitude *a*_*t*_, decay coefficient β_*t*_, and radial distance *r*. The relative phase of the effective intrinsic scatterer and the tip is Θ = 2*k*_opt_ϕ + θ + θ_*t*_*r*, where ϕ is the relative azimuthal distance, *k*_opt_ = 2*πn*_0_/λ is the optical wavenumber with refractive index *n*_0_ and vacuum wavelength of light λ, θ is the initial phase, and θ_*t*_ is a radially dependent phase accounting for the tip shape.^54^

**Figure 1:**
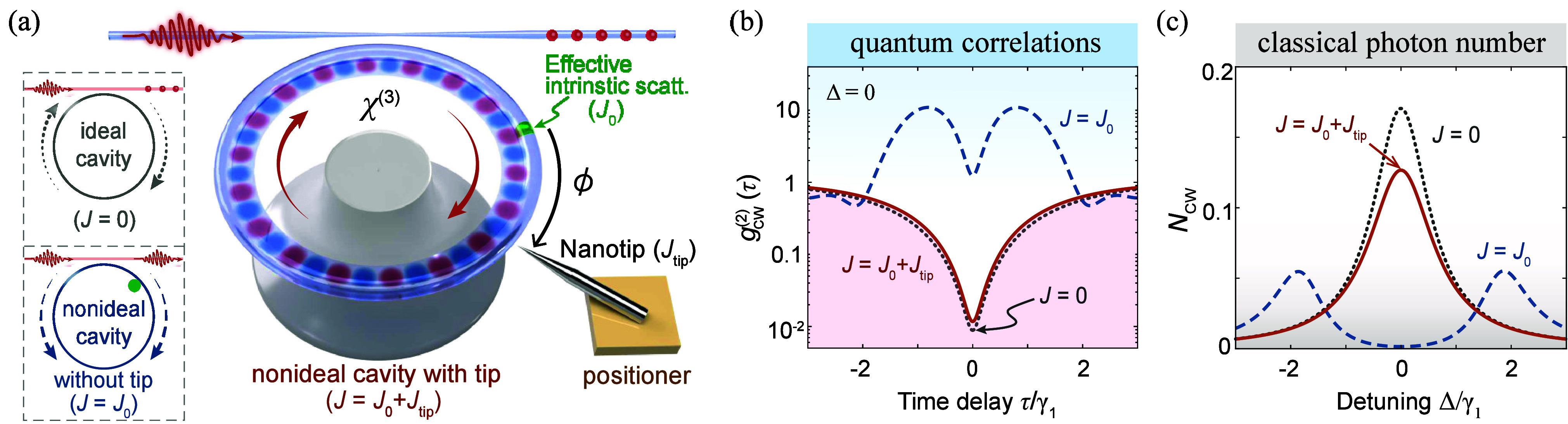
SPB against backscattering loss in a Kerr WGM cavity with an additional tip. (a) SPB occurs in an ideal nonlinear cavity (gray, *J* = 0), and is annihilated by the backscattering in a nonideal cavity (blue, *J* = *J*_0_). SPB reoccurs by tuning the relative distance ϕ between the intrinsic scatterer (*J*_0_) and the tip (*J*_tip_). (b) These effects are confirmed via the quantum correlation *g*_cw_^(2)^(τ). (c) Mean photon number *N*_cw_ versus Δ/γ_1_. Here, *J*_0_ = 1.8γ_1_ ∼ 0.4 MHz, χ/γ ∼ 5.3, ϕ = 0.27 μm. The other parameters are given in the main text.

To study SPB against backscattering loss, we consider a generic nonlinearity, Kerr nonlinearity,^10,12,77^ which was realized via light-atom couplings,^13,78^ superconducting circuits,^79^ and magnon devices,^80^ as well as theoretically studied in optomechanical systems.^81−84^ The Kerr interactions are given by^85−88^
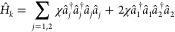
2where  is the Kerr parameter with nonlinear susceptibility χ̅^(3)^, vacuum (relative) permittivity ε_0_ (), and mode volume *V*_eff_.^85,86,89−91^ In this work, We adopt a scalar approximation for χ̅^(3)^ to estimate results at the order-of-magnitude level, neglecting the off-diagonal components of χ_*ijkl*_^(3)^ to simplify the analysis.^89,91,92^ Such Kerr interaction becomes *Ĥ*_*k*_ = *χâ*_1_^†^*â*_1_^†^*â*_1_*â*_1_ in an ideal cavity. In the frame rotating at the drive frequency ω_*L*_, the Hamiltonian of the system reads
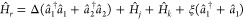
3with Δ = ω – ω_*L*_, and ω = ω_0_ + |*J*|. Here, ω_0_ is the resonance frequency of the cavity, ξ = [γ_ex_*P*_in_/(ℏω_*L*_)]^1/2^ is the driving amplitude with power *P*_in_ and cavity-waveguide coupling rate γ_ex_.

We study the classical mean photon number *N*_cw_ = ⟨*â*_1_^†^*â*_1_⟩, and the second-order quantum correlation:^93^*g*_cw_^(2)^(τ) ≡ lim_*t*→∞_[⟨*â*_1_^†^(*t*)*â*_1_^†^(*t* + τ)*â*_1_(*t* + τ)*â*_1_(*t*)⟩/⟨*â*_1_^†^(*t*)*â*_1_(*t*)⟩^2^], which is usually measured by Hanbury Brown-Twiss interferometers.^13−17^ The condition *g*_cw_^(2)^(0) < *g*_cw_^(2)^(τ) characterizes photon antibunching, and *g*_cw_^(2)^(0) ≪ 1 [or *g*_cw_^(2)^(0) ≈ 0] indicating SPB with sub-Poissonian photon-number statistics.^12,13,94,95^

This *g*_cw_^(2)^(τ) can be calculated by numerically solving the Lindblad master equation for the density operator ρ̂ of this system:^96,97^
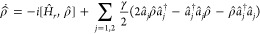
4where γ = γ_1_ + γ_tip_ is the total dissipation rate, γ_1_ = γ_0_ + γ_ex_, and γ_0_ = ω_0_/*Q* denotes the intrinsic losses of the cavity with the quality factor *Q*. The tip-induced loss is γ_tip_ = *a*_γ_exp(−2β_γ_*r*), with amplitude *a*_γ_, and decay coefficient β_γ_.^54^

The experimentally accessible parameters of the nanotip are taken as^54^*a*_*t*_ = 14.3 MHz, (2β_*t*_)^−1^ = 99 nm, θ_*t*_ = 3π/2 μm^–1^, θ = −π/2, *a*_γ_ = 2.43 MHz, and (2β_γ_)^−1^ = 92 nm. The other experimentally accessible parameters are^87,88,98−101^*V*_eff_ = 150 μm^3^, *n*_0_ = 1.4, *Q* = 10^10^, λ = 1550 nm, χ^(3)^/ε_*r*_^2^ = 1.8 × 10^–17^ m^2^/V^2^, and *P*_in_ = 4 fW. For the WGM cavities, *V*_eff_ is typically 10^2^–10^4^ μm^3^,^98,99^*Q* ∼ 10^9^–10^12^,^100,101^ and *J*_0_ ∼ 0.5 MHz–0.1 GHz.^55,56,102,103^ The Kerr coefficient for semiconductor materials with GaAs is χ^(3)^/ε_*r*_^2^ = 2 × 10^–17^ m^2^/V^2^,^87,88^ and materials with indium tin oxide reach χ^(3)^/ε_*r*_^2^ = 2.12 × 10^–17^ m^2^/V^2^.^104^ In addition, χ^(3)^ can be further enhanced to 2 × 10^–11^ m/V^2^ with other materials.^89,105^ The input power can be attenuated by passing through an electro-optic modulator, and reach to 6.3 fW.^106^ Since *P*_in_ ≪ γ, the thermal effect induced by high optical powers can be neglected.^107^ Thermal effects can also be reduced by making a thermal isolation or changing the materials of the bracket to, for instance, aluminum.^108^ In experiments, the nanotip can be fabricated on a high-purity polycrystalline tungsten wire with an electrochemical etching process, which relies on capillary action and water-based electrochemical reactions.^107^ The tip size can be precisely controlled by adjusting the etching-voltage cutoff delay. Additionally, the tungsten tip near-field probe is fixed onto a polylactic acid plastic mount, which is then attached to a computer-controlled, three-axis piezoelectric positioner. This setup allows for a positioning precision of 25 nm or even smaller.^107^ Also, realistic mechanical instability or temperature drift can be eliminated by designing a chip-based resonator with a scatterer integrated on the chip,^109^ which holds the potential for realizing backscattering-immune on-chip resonators based on microelectromechanical-systems (MEMS) techniques.

Figure 1(b) shows SPB with *g*_cw_^(2)^(0) ∼ 0.009 in an ideal Kerr cavity, since the input light fulfilling the single-photon resonance condition (Δ = 0) can only be resonant with the transition from the vacuum to the one-photon state but not with higher transitions.^12,13^ However, intrinsic defects in a nonideal cavity cause backscattering and a coupling *J*_0_ [the mode splitting in Figure 1(c)], which provides an extra path for the resonance of higher-state transitions, leading to the breakdown of SPB.

In contrast, SPB recovers with a tip [Figure 1(b)], which is also confirmed by higher-order correlations: *g*_cw_^(4)^(0) ∼ 4.8 × 10^–8^ ≪ *g*_cw_^(3)^(0) ∼ 3.6 × 10^–5^ ≪ *g*_cw_^(2)^(0) ∼ 0.012 ≪ 1. Due to the tip-induced loss, *g*_cw_^(2)^(0) is slightly larger than that in the ideal cavity. Such quantum backscattering-immune effect is different from the classical one.^51−57,59,60^

Specifically, the behavior of quantum correlation *g*_cw_^(2)^(0) depends on both of χ and ϕ, while classical photon number *N*_cw_ is independent of the χ [Figure 2]. By fixing ϕ = {0.21, 0.33} μm, the SPB can emerge with a specific strength of the nonlinearity, i.e., χ/γ = 0.5. However, *N*_cw_ is still suppressed. In addition, by fixing ϕ = 0.27 μm, the quantum revival of SPB can only exist in the strong nonlinear regime (χ/γ > 1), but cannot exist for χ/γ < 1. In contrast, the classical revival of *N*_cw_ always exists with its maximum at the same position. Different from the classical optical devices against backscattering losses rely on nanotip-induced destructive interference,^51−54^ such robust quantum effect relies on the interplay of the resonator nonlinearity and nanotip-induced optical coupling. This distinction may play the crucial role for designing robust quantum devices, where quantum fluctuations and correlations play a central role in the device performance.

**Figure 2:**
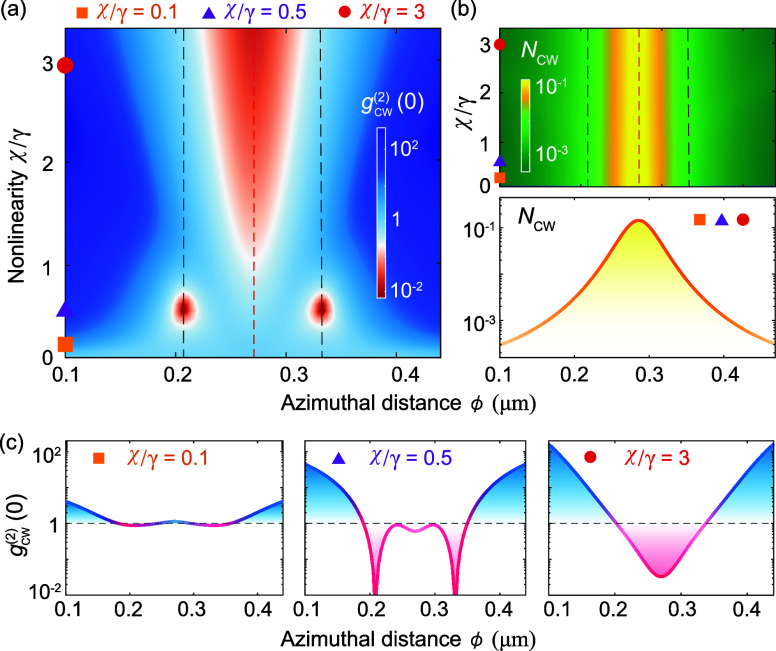
Robust quantum SPB effect is different from the behavior of classical *N*_cw_ by varying azimuthal distance ϕ and nonlinearity strengths χ/γ. (a) The quantum correlation of SPB, *g*_cw_^(2)^(0) ≪ 1, can emerge at ϕ = {0.21, 0.33} μm (vertical gray dashed lines) for χ/γ = 0.5 [purple triangles, the middle panel of (c)], while classical *N*_cw_ is still suppressed. Also, SPB cannot occur at ϕ = 0.27 μm (vertical red dashed lines) for χ/γ < 1 [orange squares, the left panel of (c)], but can occur for χ/γ > 1 [red circles, the right panel of (c)]. (b) Meanwhile the classical *N*_cw_ always recovers to its maximum at ϕ = 0.27 μm. The other parameters are the same as those in Figure 1.

The underlying physics can be understood from the interplay of the resonator nonlinearity and the tip-induced optical coupling by analyzing the photon-number probabilities and the transitions between different quantum states [Figure 3], which is different from that in previous studies in classical optics,^51−57,59,60^ i.e., merely relies on the scatterer-induced destructive interference, and focuses on light amplitudes.

**Figure 3:**
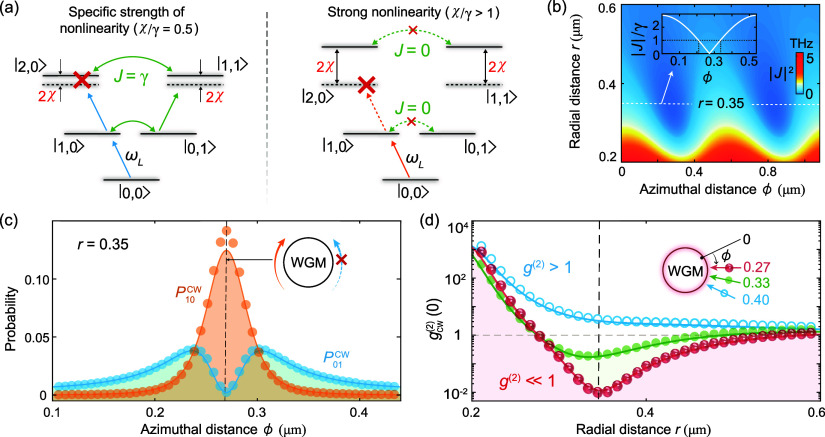
Physical mechanism of SPB can be understood from (a) the eigenenergy structures and transition paths, as well as (b) the nanotip-induced optical coupling. Specifically, for χ/γ = 0.5 [left panel in (a)], optical coupling with |*J*| = γ can be achieved at ϕ = {0.21, 0.33} μm and *r* = 0.35 μm [the inset of (b)]. SPB occurs because of the destructive interference of two transition paths (blue and green arrows) to state |2, 0⟩. For χ/γ > 1 [right panel in (b)], two optical modes decoupled (*J* = 0) at ϕ = 0.27 μm and *r* = 0.35 μm. SPB emerges due to the unequal eigenenergy spaces. Such SPB effect can also be recognized via (c) the single-photon probability distribution and (d) second-order quantum correlation. In (c, d), the curves and markers correspond to the numerical and analytical results, respectively. The Kerr nonlinearity and other parameters are the same as those in Figure 1.

We study the photon-number probabilities via the quantum trajectory method.^110^ Our effective Hamiltonian is *Ĥ*_eff_ = *Ĥ*_*r*_ – *i*∑_*j*=1,2_(γ/2)*â*_*j*_^†^*â*_*j*_. For ξ ≪ γ, by truncating the Hilbert space to *N* = *m* + *n* = 3, the states are |ψ(*t*)⟩ = ∑_*m* = 0_^3^∑_*n* = 0_^*m*^*C*_*mn*_|*m*, *n*⟩, where *C*_*mn*_ are probability amplitudes corresponding to |*m*, *n*⟩. The probability of finding *m* photons in the CW mode and *n* photons in the CCW mode is given by *P*_*mn*_ = |*C*_*mn*_|^2^, which can also be obtained from the steady-state solutions ρ_ss_ of eq 4 via *P*_*mn*_ = ⟨*m*, *n*|ρ_ss_|*m*, *n*⟩. An excellent agreement between our analytical results and numerical results is seen in Figure 3. Note that the effect of quantum jumps is ignored (considered) in the semiclassical analytical (quantum master equation) approach.^111^

For Δ = 0, the input light can be resonant with the transitions from the vacuum to |2, 0⟩ in the weak nonlinear regime (χ/γ < 1). The corresponding probability amplitude can be obtained by solving the Schrödinger equation *i*|ψ̇(*t*)⟩ = *Ĥ*_eff_|ψ(*t*)⟩:
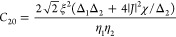
5where Δ_1_ = 2Δ – *iγ*, Δ_2_ = Δ_1_ + 2χ, η_1_ = 4|*J*|^2^ – Δ_1_^2^, and η_2_ = 4|*J*|^2^ – Δ_2_^2^. However, SPB can occur with *P*_20_ = 0,^112−114^ which can be understood from the destructive interference of two transition paths [Figure 3(a)]:  (blue), and  (green). By setting *C*_20_ = 0, the conditions of SPB are given by χ/γ = 0.5, and |*J*|/γ = 1. For *J*_0_ = 1.8γ_1_, we have *r* = 0.35 μm, and ϕ = {0.21, 0.33} μm [the inset in Figure 3(b)]. In contrast, *N*_cw_ cannot be totally revived at the same positions due to the nonzero coupling (*J* ≠ 0) between the CW and CCW modes.

The single-photon probabilities are given by
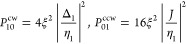
6where *P*_01_^ccw^ tends to be zero, and *P*_10_^ccw^ reaches its maximum for *J* = 0 [Figure 3(c)], i.e., *r* = ln(*a*_*t*_/*J*_0_)/2α_t_, and ϕ = [(2*l* + 1)π – θ – θ_*t*_*r*]/2*k*_opt_ with integer *l*. For *l* = {0, 1}, we have ϕ = {0.27, 0.82} μm [Figure 3(b)]. SPB emerges because the transition |0, 0⟩ → |1, 0⟩ is resonantly driven by the input light, but the transition |1, 0⟩ → |2, 0⟩ is detuned by 2χ, and the transitions between |1, 0⟩ and |0, 1⟩ are eliminated [Figure 3(a), right panel]. Such effect can be understood from the interplay of the strong nonlinearity induced unequal eigenenergy spaces (χ/γ > 1), and the tip-induced vanishing of the coupling (*J* = 0). In contrast, the classical revival of *N*_cw_ merely relies on the condition of *J* = 0, regardless of χ.

The second-order quantum correlation is given by
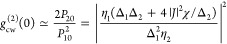
7With *J* = 0, we obtain *g*_cw_^(2)^(0) ≃ [4(χ/γ)^2^ + 1]^−1^ < 1, for χ > γ and Δ = 0, which indicates SPB [Figure 3(d)]. When the tip is away from the cavity (*r* > 0.6 μm), the system behaves as a nonideal cavity without the tip and SPB cannot be observed. When the tip is close to the cavity (0 < *r* < 0.2 μm), increased γ and *J* enable the input light to be in resonance with higher photon-number states, resulting in photon bunching.^17^

To characterize the SPB against backscattering loss efficiency, we introduce a ratio by comparing the minimum of *g*_cw_^(2)^(0) in our device (*J*_0_ ≠ 0) with that in an ideal cavity (*J*_0_ = 0) under the same optical nonlinearities and driving fields:
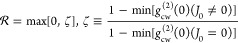
8Here, the quantity of 1–min[*g*_cw_^(2)^(0)] is the purity of the generated single photons, and  denotes perfect backscattering immunity, indicating that the single photons generated in our system with the intrinsic backscattering have the same purity as those in the ideal case. Figure 4(a) shows that the efficiency  can reach 99.7% with *J*_0_/γ_1_ = 1.8 by adjusting the nanotip position at *r* = 0.35 μm and ϕ = 0.27 μm. Furthermore, for the nonideal cavity without nanotip, such efficiency  gradually decreases with increasing *J*_0_, and becomes 0 for *J*_0_/γ_1_ = 1.8 [Figure 4(b)], i.e., SPB is suppressed by the backscattering. However, robust SPB can exist with different backscattering strenghths by introducing an additional tip with strong nonlinearities, which can be beneficial for protecting the generation or transmission of single photons, and improving the performance in realistic quantum devices.

**Figure 4:**
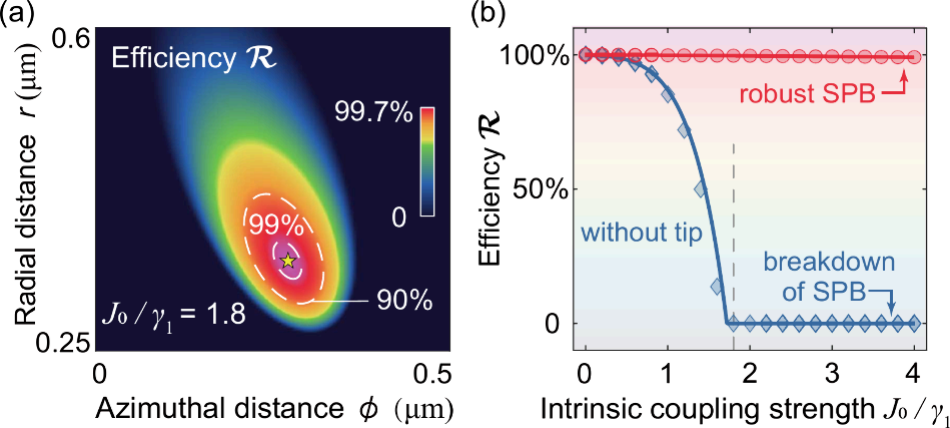
Robust SPB for different backscattering strengths. (a) SPB against backscattering loss efficiency  versus *r* and ϕ for *J*_0_/γ_1_ = 1.8 and Δ = 0. (b) Efficiency  as a function of *J*_0_ with (red) and without (blue) the nanotip, where the curves and markers show our numerical and analytical results, respectively. The Kerr nonlinearity and other parameters are the same as those in Figure 1.

We studied SPB against backscattering loss in a nonideal Kerr WGM cavity with a nanotip. The efficiency of such effect is up to 99.7% by tuning tip positions, which is robust with different backscattering strengths. More interestingly, we found that the behavior of this quantum effect is distinct from that of the classical mean-photon number with different strengths of the nonlinearity, due to the interplay of the resonator nonlinearity and the tip-induced optical coupling.

This underlying principle can be extended to other types of platforms, e.g., optical parametric amplifiers or cavity QED systems, for exploring squeezing or entanglement against backscattering loss, and for generating robust Schrödinger cat states. It is also expected to explore multiphoton bundles against backscattering loss^115−117^ or mutual blockade^15^ and robust microwave-optical photon pair^118^ by studying higher-order correlations. Our work provides a novel perspective toward enhancing the performance of quantum devices, opening a new way to protect or engineer fragile quantum resources, and holding the potential for implementations in quantum technologies, such as robust single-photon routing^119^ in quantum communications or more robust quantum sensing.^120−122^
